# Downregulated long non-coding RNA CLMAT3 promotes the proliferation of colorectal cancer cells by targeting regulators of the cell cycle pathway

**DOI:** 10.18632/oncotarget.10431

**Published:** 2016-07-06

**Authors:** Le-chi Ye, Tao Chen, De-xiang Zhu, Shi-xu Lv, Jun-jun Qiu, Jianmin Xu, Feng-Lai Yuan, Ye Wei

**Affiliations:** ^1^ Department of General Surgery, Zhongshan Hospital, Fudan University, Shanghai, P.R. China; ^2^ Department of Oncological Surgery, The First Affiliated Hospital of Wenzhou Medical University, Wenzhou, P.R. China; ^3^ Department of Gynecology, Obstetrics and Gynecology Hospital of Fudan University, Shanghai, P.R. China; ^4^ The Third Hospital Affiliated to Nantong University, Nantong, P.R. China

**Keywords:** colorectal cancer, long non-coding RNA, lncRNA-CLMAT3, cell cycle pathway

## Abstract

Over-expression of long non-coding RNA (lncRNA)-CLMAT3 is significantly associated with colorectal liver metastasis and is an independent predictor of poor survival for colorectal cancer patients. However, as little is known regarding the role of this gene in the proliferation of colorectal cancer *in vitro*, we investigated the involvement of lncRNA-CLMAT3 in colorectal cancer cell proliferation. In this study, we demonstrate that lncRNA-CLMAT3 expression was significantly increased in colorectal cancer cells compared with a normal intestinal mucous cell line and that inhibition of lncRNA-CLMAT3 suppressed colorectal cancer cell proliferation *in vitro*. We also found that this reduced colorectal cancer cell proliferation due to lncRNA-CLMAT3 knockdown is associated with G0/G1 cell-cycle arrest induction and apoptosis enhancement. Furthermore, lncRNA-CLMAT3 knockdown enhanced Cdh1 expression and resulted in p27Kip accumulation *via* increased Skp2 protein ubiquitination. Taken together, our findings suggest that reducing lncRNA-CLMAT3 inhibits colorectal cancer cell proliferation by affecting cell cycle components.

## INTRODUCTION

Colorectal cancer (CRC) is the third leading cause of death among all human malignancies. CRC causes over 600,000 deaths per year worldwide, with increased morbidity and mortality in recent years [1]. Currently, a major obstacle for CRC treatment is the high rate of growth and metastasis. The metastatic process follows a series of complex and sequential steps: (1) cancer cells successfully escape the primary tumor *via* local invasion; (2) these cells enter the lymphatic or vascular system, where they survive and translocate primarily through the bloodstream to microvessels in a distant tissue and exit from the bloodstream (extravasation); and (3) the cells grow in response to the foreign microenvironment, and this proliferation results in the formation of a macroscopic secondary tumor [2]. Metastasis is the major cause of death in many cancers, including CRC [3, 4]. However, the molecular mechanisms underlying CRC metastasis remain elusive. Therefore, a better understanding of the specific molecular mechanisms underlying CRC metastasis is required to facilitate the development of new strategies and therapeutic targets for patients with CRC.

Compared with prokaryote genomes, eukaryote genomes encode large amounts of noncoding transcripts. Among these noncoding transcripts are many long non-coding RNAs (lncRNAs), which have important functions in controlling cell proliferation, differentiation, and apoptosis, as well as epigenetic, transcriptional and post-transcriptional processes. lncRNAs, non-protein-coding transcripts longer than 200 nucleotides, account for at least 80% of the transcripts produced by the entire genome [5, 6]. Recent evidence indicates that lncRNAs have a close relationship with tumor occurrence and development and are involved in driving tumorigenesis, growth, invasion, and metastasis [6, 7]. We previously reported that a novel dysregulated lncRNA in CRC, lncRNA-colorectal liver metastasis-associated transcript 3 (CLMAT3), is highly expressed in CRC and is associated with liver metastasis [8]. We also found lncRNA-CLMAT3 expression to be a predictor of patient survival. However, the functions of lncRNA-CLMAT3 in CRC and the detailed molecular mechanisms related to these functions have not yet been elucidated.

In the current study, we focused on how lncRNA-CLMAT3 affects CRC cell proliferation by investigating its expression and the impact of suppressing lncRNA-CLMAT3, and we attempted to clarify the underlying molecular mechanism of these effects.

## RESULTS

### lncRNA-CLMAT3 expression was generally increased in human CRC cell lines

**Figure 1:**
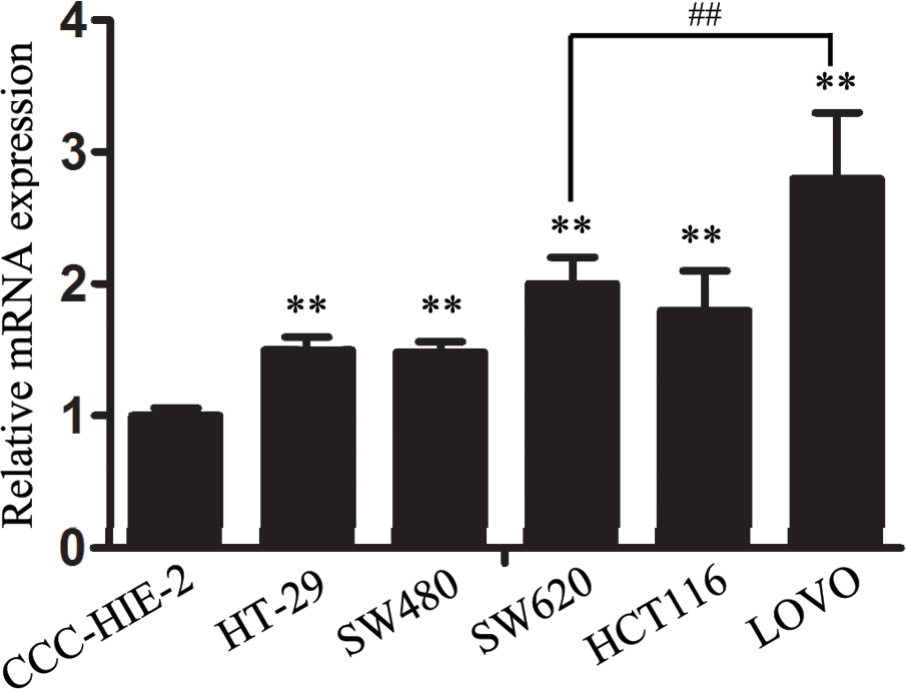
Relative lncRNA-CLMAT3 expression levels in CRC cell lines Relative lncRNA-CLMAT3 mRNA expression in CRC cell lines (HT-29, SW480, SW620, HCT116, and LOVO) and the normal intestinal mucous cell line CCC-HIE-2. **P < 0.01 compared with CCC-HIE-2, ^##^*P* < 0.01 compared with SW620.

Our previous study involving specimens from CRC patients indicated increased expression of lncRNA-CLMAT3 in these tissues, which prompted us to explore the potential impact of lncRNA-CLMAT3 deregulation on CRC progression. We therefore performed qRT-PCR analysis to examine lncRNA-CLMAT3 expression in 5 human CRC cell lines, HT-29, SW480, SW620, HCT116, and LOVOcells, as well as the normal intestinal mucous cell line CCC-HIE-2. As indicated in Figure 1, lncRNA-CLMAT3 expression in the CRC cell lines was significantly higher than that in the CCC-HIE-2 cells (*P* = 0.0004). As LOVO cells exhibited the most marked increase (Figure 1), these cells were used for lncRNA-CLMAT3 RNAi and other experiments.

### Knockdown of lncRNA-CLMAT3 in CRC cells decreased cell growth *in vitro*

Due to the abnormally high expression of lncRNA-CLMAT3 in CRC cell lines and tissues, we hypothesized that expression of this lncRNA might have an effect on CRC progression. To examine this possibility, we first silenced lncRNA-CLMAT3 expression in LOVO cells by RNA interference (RNAi). lncRNA-CLMAT3 expression was reduced upon transfection with lncRNA-CLMAT3 shRNA, indicating that this strategy was effective for specifically knocking down lncRNA-CLMAT3 expression (Figure S1).

**Figure 2:**
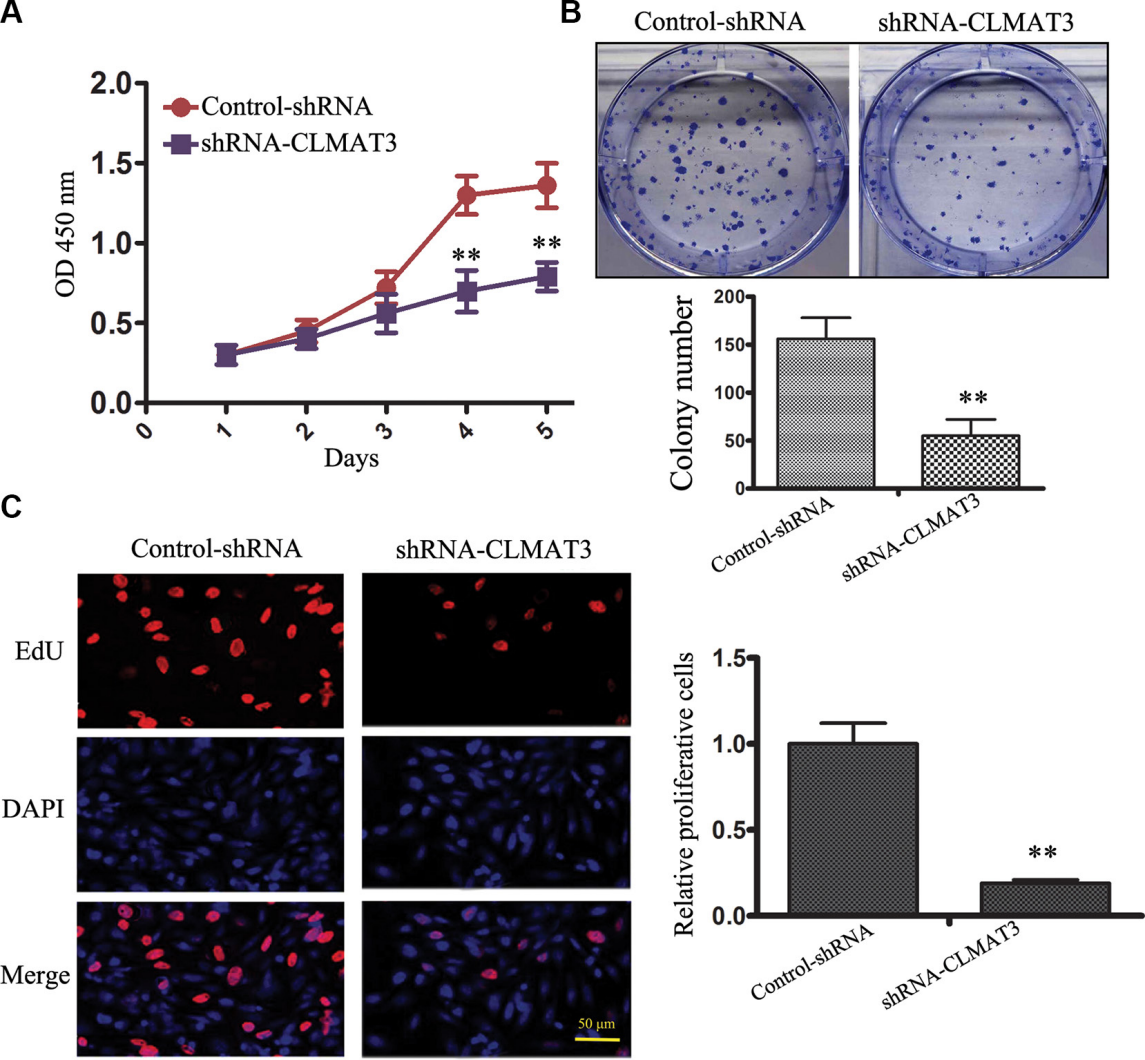
lncRNA-CLMAT3 knockdown inhibited CRC growth *in vitro* (**A**) lncRNA-CLMAT3 knockdown suppressed CRC growth *in vitro*. CCK8 assays were performed to determine the proliferation of CRC cells transfected with lncRNA-CLMAT3 shRNA and control shRNA. **P < 0.01 compared with control shRNA. (**B**) Colony-forming growth assays were also performed to assess CRC cell proliferation. The colonies were imaged and counted. **P < 0.01 compared with control shRNA. (**C**) Proliferating LOVO cells were labeled with EdU. The click-it reaction revealed EdU staining (red). The cell nuclei were stained with DAPI. The images are representative of the results obtained. **P < 0.01 compared with control shRNA.

CCK8 assays revealed that lncRNA-CLMAT3 knockdown led to inhibition of LOVO cell proliferation in a time-dependent manner, and these effects were significantly different between days 4 and 5 (Figure 2A). A colony formation assay was used to evaluate the influence of lncRNA-CLMAT3 on LOVO cell colony formation. As indicated in Figure 2B, colony formation ability was also reduced by lncRNA-CLMAT3 silencing in LOVO cells. The inhibitory effect of lncRNA-CLMAT3 knockdown on CRC proliferation was also observed by EdU (red)/DAPI (blue) immunostaining, with significantly reduced staining compared with the control group (Figure 2C). The shRNA observations indicated that low expression of lncRNA-CLMAT3 might contribute to CRC progression.

### Knockdown of lncRNA-CLMAT3 inhibited CRC cell cycle progression and induced apoptosis

**Figure 3:**
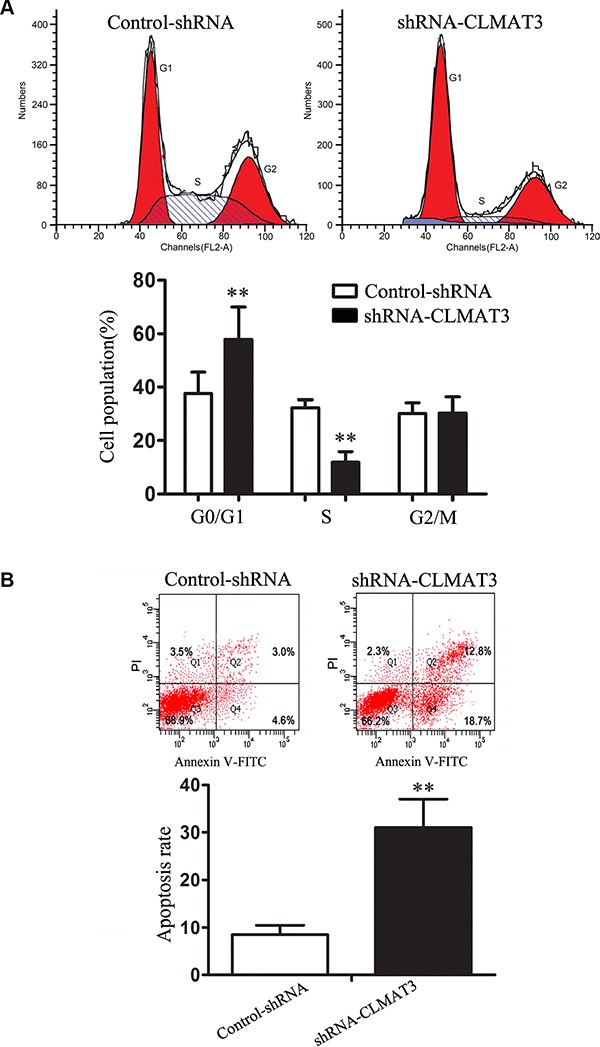
lncRNA-CLMAT3 controlled cell cycle progression and apoptosis in LOVO cells (**A**) Cell cycle analysis was performed using flow cytometry (above panel). Representative histograms are shown (below panel). **P < 0.01 compared with control shRNA. (**B**) Apoptosis was assayed by flow cytometry (above panel). The bar chart represents the sum percentage of early and late apoptotic cells (below panel). **P < 0.01 compared with control shRNA.

Cell cycle arrest reduces cell proliferation [9]. To assess whether the growth inhibitory effect of lncRNA-CLMAT3 knockdown involves changes in cell cycle progression, we treated LOVO cells with lncRNA-CLMAT3 shRNA or the empty vector for 48 h and examined the effects using flow cytometry. Compared with the control, lncRNA-CLMAT3 shRNA led to a significant accumulation of cells in G0/G1 phase and a significant decrease in S-phase cells (Figure 3A). Next, we investigated the effects of lncRNA-CLMAT3 knockdown on apoptosis. As shown in Figure 3B, the percentage of apoptotic cells was significantly increased in the lncRNA-CLMAT3 knockdown group compared with the control group. Taken together, lncRNA-CLMAT3 knockdown treatment induced apoptosis and G0/G1-phase arrest in colorectal cancer cells.

### lncRNA-CLMAT3 knockdown increased Cdh1 expression by accelerating Skp2 degradation, which resulted in p27Kip accumulation

**Figure 4:**
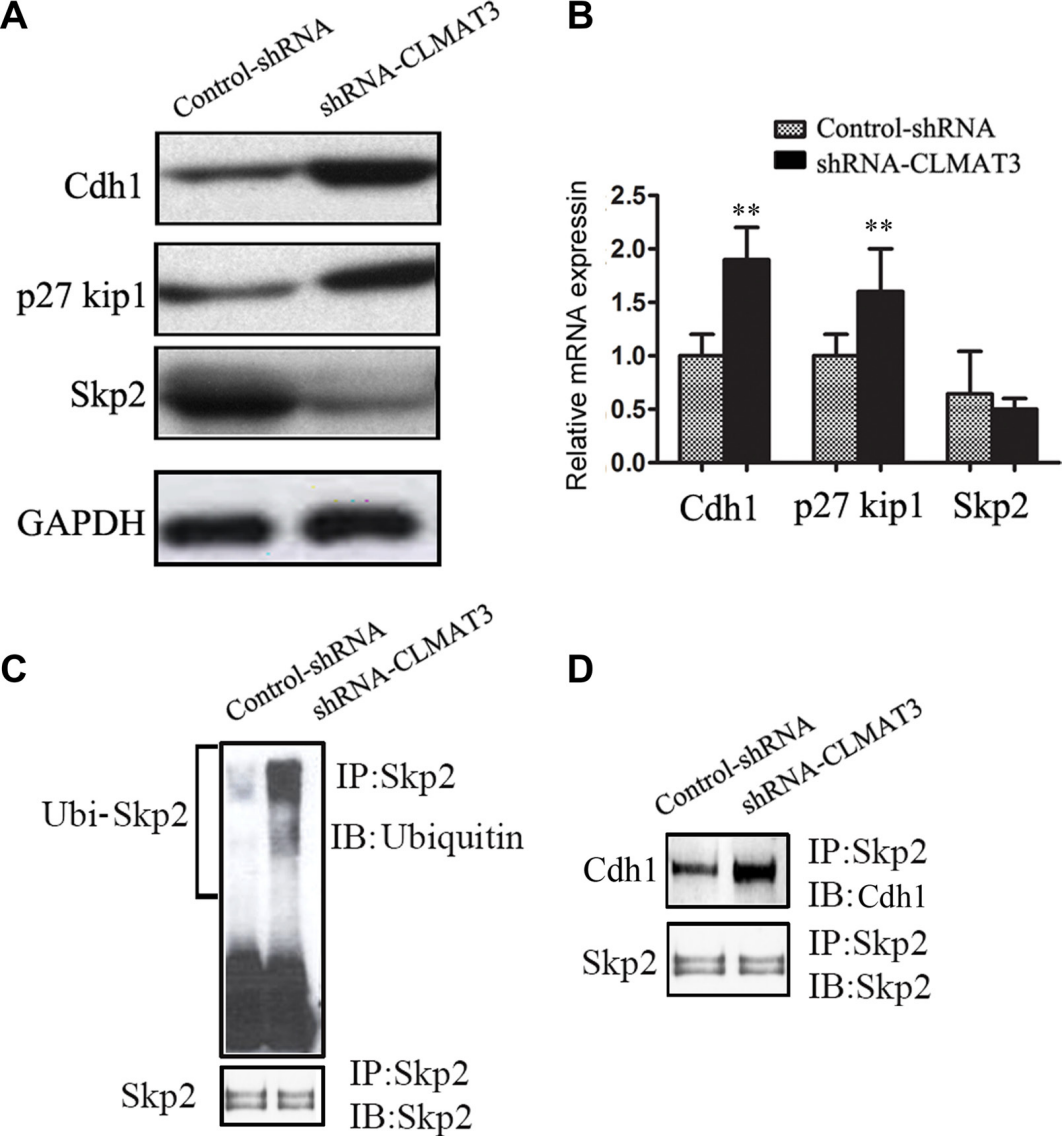
lncRNA-CLMAT3 controlled the expression of master G0/G1 regulators, including Cdh1, CDKN1B (p27Kip1) and Skp2 (**A**) Western blotting revealed the expression of Cdh1, p27Kip1, and Skp2 in LOVO cells. (**B**) The relative expression of Cdh1, p27Kip1, and Skp2 mRNA upon lncRNA-CLMAT3 knockdown was analyzed by real-time PCR. **P < 0.01 compared with control shRNA. (**C**) LOVO cell extracts were immunoprecipitated using antibodies against Skp2 or ubiquitin. Western blotting was used to detect interacting partners. (**D**) The blots were also probed with antibodies against Cdh1 to analyze interaction between Skp2 and Cdh1.

Considering that lncRNA-CLMAT3 knockdown caused cell cycle arrest at the G0/G1 phase, we used Western blotting and real-time PCR to measure the expression of master G0/G1 regulators, including Cdh1, CDKN1B (p27Kip1) and Skp2 [10, 11]. As shown in Figure 4A, Cdh1 and p27Kip1 expression was increased in the lncRNA-CLMAT3 knockdown group compared with the control group. In contrast, Skp2 exhibited lower expression levels in the lncRNA-CLMAT3 knockdown group. At the transcriptional level, Cdh1 expression was markedly increased with lncRNA-CLMAT3 knockdown (Figure 4B). Consistently, p27Kip1 mRNA expression was significantly increased in the lncRNA-CLMAT3 knockdown group compared with that in the control group (Figure 4B), though Skp2 mRNA was not significantly altered at 48 h under the same condition (Figure 4B). These data led us to hypothesize that lncRNA-CLMAT3 may directly control the Skp2 protein independently of gene transcription. This result suggests that knockdown of lncRNA-CLMAT3 directly increases the level of Cdh1 expression and causes p27Kip1 accumulation, thus resulting in Skp2 degradation.

The Skp2 protein has been identified as a target of the ubiquitin/proteasome degradation pathway *via* APC/Cdh1-controlled ubiquitination [12]. Therefore, we next determined the ubiquitination status of Skp2 and associations with Skp2 and Cdh1. LOVO cells were treated with lncRNA-CLMAT3 shRNA or the control shRNA and then lysed. The lysate was immunoprecipitated using an antibody against Skp2 and blotted with an anti-ubiquitin antibody (Figure 4). lncRNA-CLMAT3 knockdown increased Skp2 protein ubiquitination. Moreover, the binding of Cdh1 to Skp2 was also increased in lncRNA-CLMAT3-knockdown cells (Figure 3B). These results suggested that lncRNA-CLMAT3 inhibition increased Skp2 protein degradation *via* ubiquitination by APC/Cdh1.

## DISCUSSION

LncRNAs have been previously underestimated with respect to their biological significance in cancer; however, lncRNAs have recently been identified as a major feature of human CRC [13–17]. Our previous studies highlighted lncRNA-CLMAT3 based on its involvement in colorectal liver metastasis [8]. Inspired by these findings, we investigated whether lncRNA-CLMAT3 is involved in the proliferation of CRC in addition to promoting metastasis. Interestingly, the results of the present study demonstrate lncRNA-CLMAT3 over-expression in CRC. Furthermore, decreased lncRNA-CLMAT3 expression suppressed tumor proliferation *in vitro*. We then applied a flow cytometry assay to investigate the possible mechanism responsible for the effect of lncRNA-CLMAT3 in enhancing proliferation. We found that silencing lncRNA-CLMAT3 arrested the cell cycle in G0/G1 phase and promoted CRC cell apoptosis, indicating that lncRNA-CLMAT3-induced CRC cell proliferation may be associated with cell cycle and apoptosis control. Taken together, our results indicate that lncRNA-CLMAT3 promotes CRC cell proliferation *via* cell cycle arrest and apoptosis.

**Figure 5:**
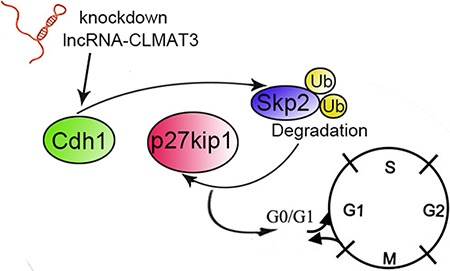
Schematic diagram of lncRNA-CLMAT3 regulation of Cdh1 expression The effects of lncRNA-CLMAT3 promote p27Kip1 accumulation by accelerating Skp2 degradation, which subsequently results in cell cycle arrest at the G0/G1 phase.

Anaphase-promoting complex (also called the cyclosome o APC/C) is a ubiquitin protein ligase that, together with Cdc20 and Cdh1, acts on distinct substrates at specific stages during the cell cycle [18]. Some studies have indicated that Cdh1 is required for suppressing proliferation in specific progenitor cells and mammalian cells [11]. Activation of the APC/Cdh1–Skp2–p27Kip1 pathway causes degradation of Skp2 and therefore accumulation of the cell cycle inhibitor protein p27Kip1, which induces G0/G1-stage cell cycle arrest [19]. Liu et al. [20] demonstrated that imatinib mesylate inhibits gastrointestinal stromal tumor cell proliferation through the Cdh1–Skp2–p27Kip1 pathway. Consistent with these previous studies, we found that lncRNA-CLMAT3 knockdown increased Cdh1 expression by accelerating Skp2 degradation, resulting in p27Kip accumulation. These results demonstrate that lncRNA-CLMAT3 knockdown induces cell cycle arrest and apoptosis by affecting the APC/Cdh1–Skp2–p27Kip1 signaling axis (Figure 5).

In summary, we demonstrated that lncRNA-CLMAT3 expression is increased in CRC cells. The effects of lncRNA-CLMAT3 on cell proliferation suggest that it promotes tumorigenesis in CRC. Finally, we demonstrated that lncRNA-CLMAT3 controls cell proliferation *via* cell cycle arrest at the G0/G1 phase. lncRNA-CLMAT3 knockdown enhanced Cdh1 expression and p27Kip1 accumulation, causing cell cycle arrest at the G0/G1 phase by increasing Skp2 degradation in CRC cells. These results suggest that lncRNA-CLMAT3 may serve as a novel target for controlling cell cycle progression and thus an effective target for CRC therapy. Although our data demonstrate that decreased lncRNA-CLMAT3 inhibits CRC cell proliferation *in vitro*, further studies are warranted to comprehensively explore the role of lncRNA-CLMAT3 in animal experiments and in patients with CRC.

## MATERIALS AND METHODS

### Cell lines

Human CRC cell lines HT-29, SW480, SW620, HCT116, LOVO, and CCC-HIE-2 were cultured in Dulbecco's Modified Eagle Medium (DMEM; Gibco, Grand Island, NY, USA) supplemented with 10% fetal bovine serum (FBS; Gibco). All cells were cultured at 37°C in an atmosphere of 5% CO_2_.

### Quantitative reverse transcription-polymerase chain reaction (qRT-PCR)

Total RNA was extracted from human CRC cell lines using the TRIzol reagent (Invitrogen, California, USA) according to the manufacturer's instructions. Total RNA (1 μg) was reverse transcribed using M-MLV reverse transcriptase (Fermentas, USA), following the manufacturer's protocol. The PCR primers are listed in Table S1. Quantitative RT-PCR was then performed using SYBR Green Dye Detection of Amplification Products (Applied Biosystems, Foster City, CA, USA) with a GeneAmp 5700 Sequence Detection System following the protocol of the supplier. The fold change in target mRNA was calculated using the 2-ΔΔCt method after normalization to β-actin.

### Lentiviral vectors for lncRNA-CLMAT3 short hairpin RNA

Lentivirus (GV118)-encoding short hairpin RNA (shRNA) targeting lncRNA-CLMAT3 was prepared by GenePharma (Shanghai, China). The interfering sequences corresponding to distinct regions of lncRNA-CLMAT3 are listed in Table S2. A negative control shRNA was also synthesized by GenePharma. LOVO cells were plated in 6-well plates at a density of 1 × 10^4^ cells/well and incubated for 12 h at 37°C. The culture medium was then replaced with fresh DMEM, and the cells were immediately infected with shRNA lentiviruses at a multiplicity of infection (MOI) of one (2 μL). After 24 h of growth at 37°C, the medium was replaced with fresh medium; the cells were cultured for 96 h at 37°C and then harvested for staining. The knockdown efficiency at the mRNA level was assessed using quantitative RT-PCR.

### Cell proliferation assay

Cells seeded in 96-well plates at a density of 5 × 10^3^ cells per well were transfected with lncRNA-CLMAT3 shRNA or control shRNA, and cell proliferation assays were performed every 24 h using a Cell Counting Kit-8 (Sigma) per the manufacturer's protocol. The number of viable cells was measured by their absorbance at 450 nm at the indicated time points.

### 5-Ethynyl-2′-deoxyuridine (EdU) assays

Proliferation of CRC cells was also monitored using a Cell-Light EdU DNA cell proliferation kit (Ribo Bio, Guangzhou, China) according to the manufacturer's instructions. LOVO cells were seeded in 96-well plates and transfected with lncRNA-CLMAT3 shRNA or the negative control. After 48 h, the cells were stained with 50 μM EdU for 2 h. The cell nuclei were stained with DAPI for 1 h, and the cells were then examined using a florescence microscope (Olympus, Tokyo, Japan).

### Clonogenic assay

For the colony formation assay, LOVO cells transfected with lncRNA-CLMAT3 shRNA or control shRNA were plated in 6-well plates at a density of 500/well and maintained for 12 days in a medium containing 10% FBS to allow colony formation, replacing the medium every 3 days. The colonies were subsequently fixed with methanol and stained using 1% crystal violet in 70% ethanol. Visible colonies were counted manually.

### Flow cytometric analysis of the cell cycle and apoptosis

LOVO cells transfected with the shRNA targeting lncRNA-CLMAT3 or empty vector were cultured in 6-well plates for 48 h, washed with ice-cold phosphate-buffered saline (PBS), and fixed in cold 70% ethanol overnight at –20°C. The cells were then washed and stained with propidium iodide (PI, Beyotime, China) containing 0.25 mg/mL of RNase A. After incubation in the dark at 4°C for 15 min, the cells were analyzed by flow cytometry (FACScan; BD Biosciences) using MODFIT software. The percentages of various cell cycle phases (G0/G1, S, and G2/M phases) were counted and compared.

Apoptosis was examined using an AnnexinV-fluorescein isothiocyanate (FITC)/PI dual staining kit (KeyGen, Nanjing, China). Briefly, LOVO cells were treated with lncRNA-CLMAT3 shRNA or the control for 48 h and then washed three times in cold PBS. The cells were stained with AnnexinV-FITC and PI in 500 μL of binding buffer in the dark at room temperature for 15 min and quantified by flow cytometry.

### Western blotting

The cells in each group were lysed in RIPA lysis buffer (150 mM NaCl, 1.0% Nonidet P-40, 0.5% deoxycholate, 0.1% sodium dodecyl sulfate (SDS), 50 mM Tris, 20 mM glucose, pH 7.4) supplemented with the protease inhibitor phenylmethanesulfonyl fluoride (PMSF; Roche Molecular Biochemicals, Indianapolis, IN, USA). The protein concentration was measured using a BCA-200 Protein Assay kit (Pierce, Rockford, USA). The lysates (20 μg) were resolved by 4–20% gradient sodium dodecyl sulfate polyacrylamide gel electrophoresis (SDS-PAGE) and transferred to Immobilon polyvinyl difluoride membranes. After transfer, the membranes were blocked with 5% BSA at room temperature for 1 h. Primary antibodies against the following were used: Cdh1 (Calbiochem at 1:1000), Skp1 (Santa Cruz, 1:2000) and p27 (Santa Cruz, 1:3000). The membranes were incubated with secondary antibodies (Santa Cruz, CA, 1:1000) at room temperature for 2 h.

### Immunoprecipitation

LOVO cells transfected with the shRNA targeting lncRNA-CLMAT3 or empty vector were cultured in 6-well plates for 48 h, washed with ice-cold PBS and lysed in RIPA buffer. The lysates were centrifuged at 12,000 rpm for 5 min and incubated in a 1 mL volume with anti-Skp2, -ubiquitin, and -Cdh1 antibodies overnight at 4°C. Protein A/G-conjugated beads (20 μL) were added and incubated for 12 h. The beads were washed three times with RIPA lysis buffer. After centrifugation, the sedimented protein was used for Western blotting.

### Statistical analysis

Data are presented as the mean ± standard deviation (SD). Statistical analysis was performed using SPSS 13.0 software. Differences between groups were analyzed using Student's *t*-test when comparing only two groups or one-way analysis of variance when comparing more than two groups. *P*-values < 0.05 were considered to be significant.

## SUPPLEMENTARY MATERIALS FIGURE AND TABLES



## References

[1] Siegel R, Naishadham D, Jemal A (2012). Cancer statistics, 2012. CA Cancer J Clin.

[2] Chaffer CL, Weinberg RA (2011). A perspective on cancer cell metastasis. Science.

[3] Fidler IJ (2003). The pathogenesis of cancer metastasis: the ‘seed and soil’ hypothesis revisited. Nat Rev Cancer.

[4] Zhao Y, Miao G, Li Y, Isaji T, Gu J, Li J, Qi R (2014). MicroRNA- 130b suppresses migration and invasion of colorectal cancer cells through downregulation of integrin beta1 [corrected]. PLoS One.

[5] Ponting CP, Oliver PL, Reik W (2009). Evolution and functions of long noncoding RNAs. Cell.

[6] Hauptman N, Glavac D (2013). Long non-coding RNA in cancer. Int J Mol Sci.

[7] Zhang H, Chen Z, Wang X, Huang Z, He Z, Chen Y (2013). Long non-coding RNA: a new player in cancer. J Hematol Oncol.

[8] Ye LC, Ren L, Qiu JJ, Zhu DX, Chen T, Chang WJ, Lv SX, Xu J (2015). Aberrant expression of long noncoding RNAs in colorectal cancer with liver metastasis. Tumour Biol.

[9] Ding J, He G, Gong W, Wen W, Sun W, Ning B, Huang S, Wu K, Huang C, Wu M, Xie W, Wang H (2009). Effects of nickel on cyclin expression, cell cycle progression and cell proliferation in human pulmonary cells. Cancer Epidemiol Biomarkers Prev.

[10] Skaar JR, Pagano M (2008). Cdh1: a master G0/G1 regulator. Nat Cell Biol.

[11] Garcia-Higuera I, Manchado E, Dubus P, Canamero M, Mendez J, Moreno S, Malumbres M (2008). Genomic stability and tumour suppression by the APC/C cofactor Cdh1. Nat Cell Biol.

[12] Chen JY, Wang MC, Hung WC (2011). Bcr-Abl-induced tyrosine phosphorylation of Emi1 to stabilize Skp2 protein via inhibition of ubiquitination in chronic myeloid leukemia cells. J Cell Physiol.

[13] Xie X, Tang B, Xiao YF, Xie R, Li BS, Dong H, Zhou JY, Yang SM (2016). Long non-coding RNAs in colorectal cancer. Oncotarget.

[14] Ye LC, Zhu X, Qiu JJ, Xu J, Wei Y (2015). Involvement of long non-coding RNA in colorectal cancer: from benchtop to bedside (Review). Oncol Lett.

[15] Yang L, Qiu M, Xu Y, Wang J, Zheng Y, Li M, Xu L, Yin R (2016). Upregulation of long non-coding RNA PRNCR1 in colorectal cancer promotes cell proliferation and cell cycle progression. Oncol Rep.

[16] Shi J, Li X, Zhang F, Zhang C, Guan Q, Cao X, Zhu W, Zhang X, Cheng Y, Ou K, Chen Q, Hu S (2015). Circulating lncRNAs associated with occurrence of colorectal cancer progression. Am J Cancer Res.

[17] Ma Y, Yang Y, Wang F, Moyer MP, Wei Q, Zhang P, Yang Z, Liu W, Zhang H, Chen N, Wang H, Qin H (2015). Long non-coding RNA CCAL regulates colorectal cancer progression by activating Wnt/beta-catenin signalling pathway via suppression of activator protein 2alpha. Gut.

[18] Arnold L, Hockner S, Seufert W (2015). Insights into the cellular mechanism of the yeast ubiquitin ligase APC/C-Cdh1 from the analysis of *in vivo* degrons. Mol Biol Cell.

[19] Chen CF, Dou XW, Liang YK, Lin HY, Bai JW, Zhang XX, Wei XL, Yao-Chen L, Zhang GJ (2015). Notch3 overexpression causes arrest of cell cycle progression by inducing Cdh1 expression in human breast cancer cells. Cell Cycle.

[20] Liu Y, Perdreau SA, Chatterjee P, Wang L, Kuan SF, Duensing A (2008). Imatinib mesylate induces quiescence in gastrointestinal stromal tumor cells through the CDH1-SKP2-p27Kip1 signaling axis. Cancer Res.

