# Supplementary material for: Downregulated long non-coding RNA CLMAT3 promotes the proliferation of colorectal cancer cells by targeting regulators of the cell cycle pathway
Source: Oncotarget. 2016 Jul 6;7(37):58931–8. doi: 10.18632/oncotarget.10431 (PMC5312286; doi:10.18632/oncotarget.10431)
Supplement: Supplementary file 1 [file oncotarget-07-58931-s001.pdf]

Downregulated long non-coding RNA CLMAT3 promotes the proliferation of colorectal cancer cells by targeting regulators of the cell cycle pathway

Supplementary Materials

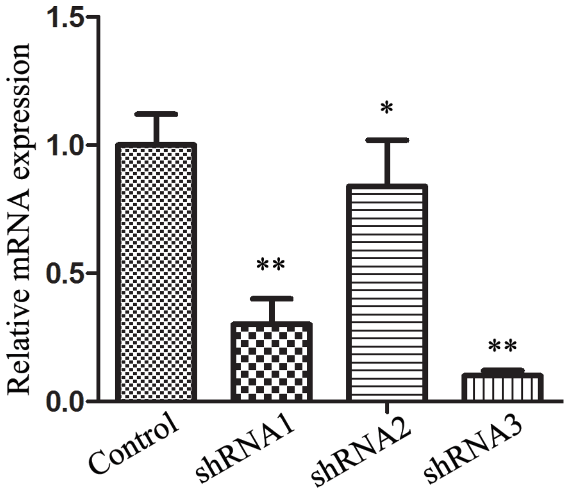

Supplementary Figure S1: Quantitative PCR detection lncRNA-CLMAT3 expression.

Supplementary Table S1: Primer sequences for lncRNA-CLMAT3

Gene		Sequences (5′–3′)
lncRNA-CLMAT3	Forward	TCCTTGACCTTTGGATTTGG
	Reverse	CGACAGAATAACCCGTGGAA
GAPDH	Forward	TGACTTCAACAGCGACACCCA
	Reverse	CACCCTGTTGCTGTAGCCAAA

Supplementary Table S2: shRNA used for silencing the lncRNA-CLMAT3

shRNA	Sequence
lncRNA-CLMAT3	5′-GATCC**GAAATGCCATAGCCTGCAAT**ATTCAAGAGATATTGCAGGCTATGGC ATTTCTTTTTTG -3′
	5′-AATTCAAAAAA**GAAATGCCATAGCCTGCAATA**TCTCTTGAATATTGCAGGCT ATGGCATTTCG -3′
Negative control shRNA	5′-GATCCGTTCTCCGAACGTGTCACGTTTCAAGAGAACGTGACACGTTCGGA GAACTTTTTTG-3′
	5′-AATTCAAAAAAGTTCTCCGAACGTGTCACGTTCTCTTGAAACGTGACACGT TCGGAGAACG-3′
